# Managing Fluid Retention in Terminal Pancreatic Cancer: The Effect of Discontinuing Intravenous Fluids

**DOI:** 10.7759/cureus.110165

**Published:** 2026-06-03

**Authors:** Takayoshi Tsubaki

**Affiliations:** 1 Department of Internal Medicine, Kimura Hospital, Medical Corporation Shishokai, Fukui, JPN

**Keywords:** ascites, cachexia, cancer, edema, infusion, intravenous therapy, palliative care, pleural fluid, quality of life

## Abstract

Intravenous fluids are often administered to maintain hydration during clinical management of advanced cancer. However, their effectiveness and safety at the terminal phase remain a subject of rigorous debate. The physiological ability to metabolize artificial hydrates is frequently compromised among patients with irreversible cachexia associated with terminal cancer. Consequently, administering excessive fluids to such patients can lead to worsening limb edema and pleural effusion accumulation or ascites. This can increase discomfort and suffering among patients, and significantly reduce their quality of life.

An 86-year-old woman was diagnosed with pancreatic head cancer with liver metastases and was initially referred for treatment of a cerebral infarction. She remained hospitalized under palliative symptom management and end-of-life care. She initially received intravenous fluids along with oral intake, but progressive lower limb edema developed and she rapidly gained weight. Following a discussion with the patient and her family, intravenous fluid therapy was discontinued, and care focused on supporting her current oral intake. Consequently, the lower limb edema improved, and the patient was able to remain hospitalized without distressing symptoms such as dyspnea.

This case suggests that the clinical benefits of intravenous fluids should be carefully balanced against the risk of exacerbating physical distress when patients with terminal cancer have worsening fluid retention. Our findings align with current palliative care insights, indicating that prioritizing comfort-oriented care can lead to a more peaceful end-of-life experience.

## Introduction

Intravenous fluids are frequently administered to hospitalized patients. However, patients with advanced cancer often develop metabolic disturbances such as irreversible cachexia associated with disease progression, and excessive nutritional therapy can impose an additional physiological burden [1]. Excessive fluid administration can promote fluid retention, leading to generalized edema, increased pleural effusion, and ascites [2]. Currently, the management of fluid therapy in patients with terminal cancer has not reached consensus among healthcare professionals [3]. Consequently, patients with terminal cancer might not receive optimal palliative care and be deprived of meaningfully spending their remaining time.

This case study describes a female patient with pancreatic cancer and liver metastases who was initially administered intravenous fluids. However, these were discontinued because she developed fluid retention and weight gain. She was maintained by oral intake; the fluid retention was reduced, and she had no signs of discomfort.

As a result, the patient was able to spend the remainder of her life peacefully, without any worsening of symptoms. My findings highlight the importance of a flexible symptom-oriented approach to hydration in palliative care settings.

This article was previously presented as a poster at the 41st Annual Meeting of the Japanese Society for Parenteral and Enteral Nutrition Therapy on February 13, 2026.

## Case presentation

An 86-year-old woman who presented at a clinic with a primary complaint of left-sided muscle weakness, blood pressure of 188/90 mmHg, and a positive Barré sign in the left upper limb was referred to our hospital. Although the muscle weakness gradually improved, a central nervous system disorder was suspected, and she was referred to our hospital. Her medical history included myocardial infarction (postpercutaneous coronary intervention), paroxysmal atrial fibrillation, and Alzheimer’s disease. Brain MRI revealed multiple small acute infarcts in the cerebellum (Figure 1A, red arrow) and cerebral cortex (Figure 1B, red arrow); the left vertebral artery was not visualized (Figure 1C, red arrows), and the left posterior cerebral artery was poorly visualized (Figure 1D, red arrow). The patient was immediately admitted to our hospital for treatment.

**Figure 1:**
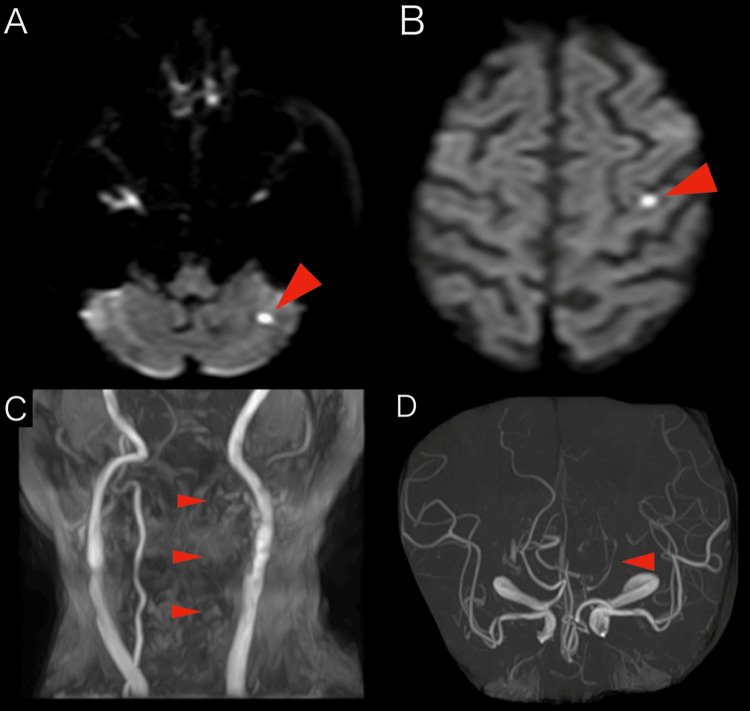
Magnetic resonance imaging findings at initial visit Infarctions in (A) the cerebellum (red arrow) and (B) the cerebral cortex (red arrow). (C) The left vertebral artery is invisible (red arrows), and (D) the left posterior cerebral artery is poorly visualized (red arrow)

A physical examination revealed the following: height, 144.5 cm; body weight, 41.6 kg; pulse, 64 beats/minute; blood pressure, 130/54 mmHg; and body temperature, 36.1℃. Edema was absent, the left muscle weakness improved, and she could clench her fists. Blood tests upon admission revealed elevated bilirubin and hepatobiliary enzymes (Table 1). She was conservatively administered with intravenous edaravone (30 mg twice daily) to treat the cerebral infarction.

**Table 1:** Laboratory data at the time of admission ALP, alkaline phosphatase; ALT, alanine aminotransferase; AST, aspartate aminotransferase; BUN, blood urea nitrogen; CRP, C-reactive protein; LD, lactate dehydrogenase; γ-GT, γ-glutamyl transferase

Laboratory test	Result	Normal range
White blood cell count	68.8	31.7-84.0 × 10^2^/µL
Hemoglobin	10.5	11.0-14.7 g/dL
Platelet count	34.3	16.7-39.0 × 10^4^/µL
CRP	3.66	0-0.14 mg/dL
BUN	10.2	8.0-20.0 mg/dL
Creatinine	0.61	0.47-0.79 mg/dL
Total protein	7.0	6.5-8.3 g/dL
Albumin	3.2	3.8-5.3 g/dL
Cholinesterase	165	213-501 U/L
Total bilirubin	1.4	0.2-1.2 mg/dL
AST	78	8-38 U/L
ALT	79	4-43 U/L
ALP	602	38-113 U/L
γ-GT	710	0-48 U/L
LD	200	124-222 U/L

The patient reported generalized fatigue on hospital day (HD)16, and blood findings revealed further elevated bilirubin and hepatobiliary enzyme values. Table 2 shows the timeline of blood tests on or after HD16.

**Table 2:** Laboratory data on HD 16-87 ALP, alkaline phosphatase; ALT, alanine aminotransferase; AST, aspartate aminotransferase; LD, lactate dehydrogenase; γ-GT, γ-glutamyl transferase; HD, hospital day

Laboratory test	16	29	43	57	72	87	Normal range
Total protein	6.1	5.1	5.4	5.1	5.5	5.4	6.5-8.3 g/dL
Albumin	2.3	1.8	1.7	1.7	1.8	1.7	3.8-5.3 g/dL
Cholinesterase	125	120	125	112	103	91	213-501 U/L
Total bilirubin	3.6	3.0	9.2	9.2	14.4	15.5	0.2-1.2 mg/dL
AST	124	100	108	129	93	74	8-38 U/L
ALT	81	34	49	95	74	53	4-43 U/L
ALP	641	911	773	1282	730	643	38-113 U/L
γ-GT	704	965	637	1743	412	298	0-48 U/L
LD	242	232	250	234	281	257	124-222 U/L

The following findings were obtained from chest and abdominal CT images acquired on the same day. A mass with a maximum diameter of ~23 mm was located within the red circle in the pancreatic head (Figure 2A, red circle). Furthermore, the common and intrahepatic bile ducts were dilated (Figure 2B, red arrow), and liver metastases were identified in segment 8 (Figure 2B; yellow arrow). Bilateral pleural effusions were also found (Figure 2C, red arrows), but without ascites. The patient was diagnosed with pancreatic head cancer with liver metastases. Considering the age of the patient and underlying medical conditions, active treatment was not pursued, and best supportive care was applied.

**Figure 2:**
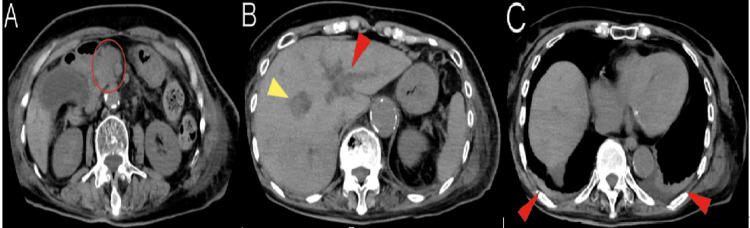
CT findings at the time of diagnosis of pancreatic cancer (A) Mass with a maximum diameter of ~23 mm in the pancreatic head is consistent with cancer (red circle). (B) Dilated common and intrahepatic bile ducts (red arrow). Mass in segment 8 of the liver is consistent with liver metastasis (yellow arrow). (C) Bilateral pleural effusions (red arrows)

Because the oral intake of the patient was unstable at the time of diagnosis, intravenous lactated Ringer’s solution (1,000 mL/day) was administered on HD17. However, the patient began to gain weight (Figure 3A), and lower leg edema became more obvious (Figure 3B). Intravenous fluid administration was reduced to 500 mL/day on HD38, but she continued to gain weight, and fluid retention appeared to progress. Consequently, intravenous fluid administration was discontinued on HD51. The patient’s family immediately started bringing lunch (Mondays and Thursdays) and dinner (Tuesdays, Wednesdays, and weekends). Despite variations in oral intake, the patient generally consumed all meals with family assistance and began to lose weight (Figure 3A). In addition, wrinkling on the tops of both feet (Figure 3C) suggested improved fluid retention.

**Figure 3:**
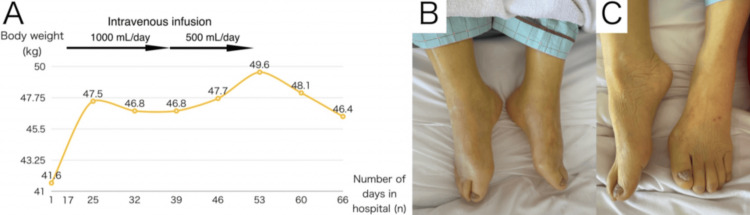
Intravenous fluid administration, changes in body weight, and lower limb findings during hospitalization (A) Patient gained and lost weight on HD17 and 51 when intravenous fluids were reinitiated and discontinued, respectively. (B) Bilateral lower limb edema worsened during this period. (C) After the discontinuation of intravenous fluids, bilateral lower limb edema decreased, and wrinkling became visible HD, hospitalization day

The patient vomited on HD72, and abdominal CT images revealed an enlarged primary tumor (Figure 4A, red circle), and further dilation of the intrahepatic bile ducts (Figure 4B, red arrows) indicated disease progression. However, no gastric distension (Figure 4B, yellow arrow) or small bowel strictures were found (Figure 4C). Ascites was scant, and evidence of peritoneal dissemination was absent (Figure 4C).

**Figure 4:**
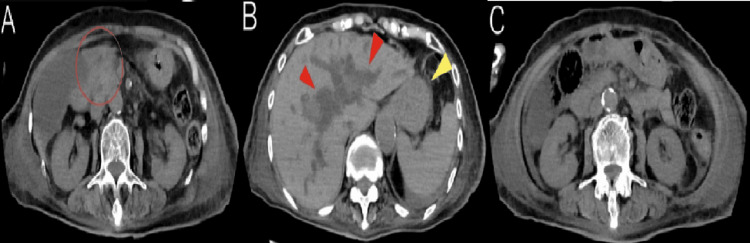
Abdominal CT findings on HD72 (A) The primary tumor is enlarged (red circle). (B) Intrahepatic bile ducts are dilated (red arrows), but gastric dilation is not evident (yellow arrow). (C) Minimal ascites and small bowel strictures are not evident HD, hospitalization day

Oral intake was maintained because signs of intestinal obstruction were not apparent. Her oral intake gradually decreased, and she received only nutritional supplements on HD82. Her family continued to bring meals, and she was able to eat until two days before she passed away peacefully, surrounded by family members. Fluid retention had not worsened, distressing symptoms such as dyspnea had not developed, and blood tests showed a downward trend in hepatobiliary enzyme levels.

## Discussion

Nutritional support via intravenously administered fluid therapy is a popular clinical intervention for hospitalized patients when a limited prognosis is anticipated and oral intake is insufficient to maintain a reasonable quality of life [4,5]. However, when the disease progresses to the terminal stage, as in our patient, cachexia becomes irreversible, and it causes severe metabolic dysfunction [6] and leads to generalized edema, ascites, and pleural effusions that are difficult to control [2]. Excessive nutritional supplementation does not improve the health-related quality of life and can impose a physiological barrier after cachexia becomes irreversible [7-9]. Excessive fluid administration leads to fluid retention, which can significantly burden patients [1,2].

We initially administered intravenous fluids to our patient to provide nutritional support because her oral intake was insufficient when she was initially diagnosed with cancer. However, the lower limb edema worsened, and she soon gained weight. The intravenous fluids were reduced, but she did not lose weight. When oral intake was maintained with the support of her family, intravenous fluids were discontinued, which stopped the progressive fluid retention, and the patient started to lose weight. The patient had no signs of distress such as dyspnea and remained comfortable during her final days.

Peritoneal dissemination is prevalent in pancreatic cancer, and it recurs in 14% of patients at the time of the initial diagnosis and in 50% of those at the time of death [10]. Malignant tumors account for 13%-20% of small bowel obstructions [11]. However, our patient did not have obvious metastases or intestinal obstructions. The amount of ascites was minimal, and intestinal motility was preserved. The absence of intestinal obstructions and the strong family support enabled the patient to maintain a fulfilled life.

Previous reports have suggested that fluid therapy for end-of-life patients may help prevent delirium and alleviate dry mouth in cancer patients with a limited life expectancy, thereby maintaining quality of life without exacerbating physical symptoms, as well as potentially reducing the psychological burden on family members providing care [5,12,13]. However, alongside these benefits, there are also reports pointing out disadvantages, such as the fact that parenteral fluid therapy does not affect survival time and is associated with fluid retention (such as pleural and ascites effusions and edema of the extremities) and an increased incidence of dyspnea and nausea, leading to concerns about a decline in the quality of end-of-life care [9,14].

Although guidelines for fluid therapy in patients with advanced terminal cancer have not been standardized [8,15], it has also been suggested that this could enable cancer patients to prepare for the final stages of their illness and die peacefully [16]. This is a single-case report and no definitive conclusions can be drawn. Nevertheless, this study suggested that adjusting fluid therapy in the context of physical symptoms during the terminal phase of life might be beneficial for patients with terminal cancer.

## Conclusions

Patients in the terminal stages of cancer develop cachexia, which is characterized by metabolic disturbances that lead to fluid retention such as limb edema, accumulation of pleural effusions, and ascites. This can lead to physical discomfort and might contribute to reducing the quality of life among patients. A standardized policy on whether to discontinue or continue intravenous therapy is difficult to establish because it depends on the status of individual patients. Therefore, treatment should be decided on a case-by-case basis. Clinicians should carefully evaluate the value of intravenous therapy to help patients pass away peacefully and comfortably.
